# Supplementary material for: Synchronous high-amplitude co-fluctuations of functional brain networks during movie-watching
Source: Imaging Neurosci (Camb). 2023 Nov 7;1:imag-1-00026. doi: 10.1162/imag_a_00026 (PMC12219993; doi:10.1162/imag_a_00026)
Supplement: Supplementary Material [file imag_a_00026-supp.pdf]

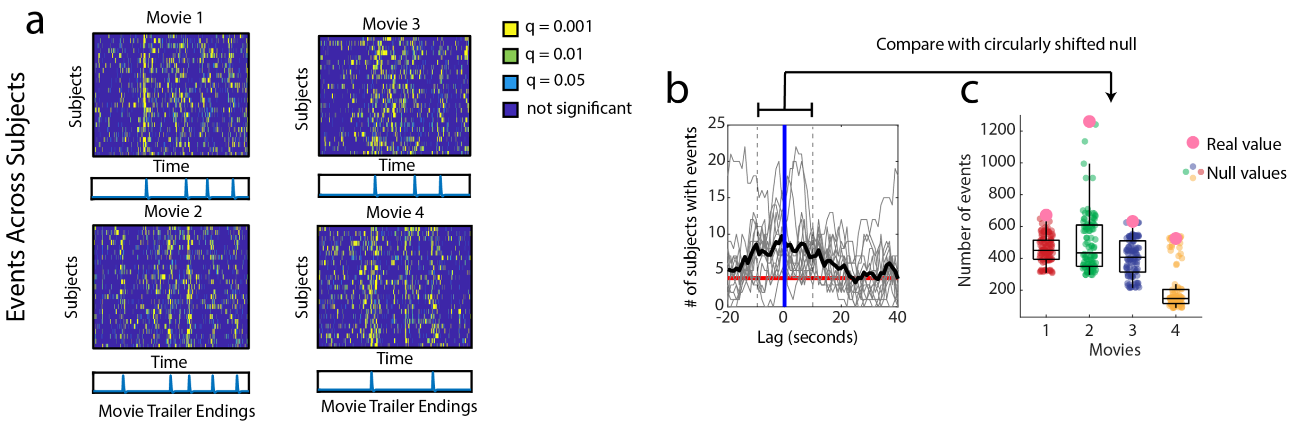

FIG. S1. **Replication of synchronous boundary events$_1$ in Indiana University data set.** (*a*) Each panel represents two things: 1) the stacked event time series of all subjects, and 2) the HRF-convolved movie trailer endings. Each of the four figures is representative of a different scan/movie-presentation for the Indiana University data set. (*b*) Plot showing the number of subjects with an event for each trailer ending (for every scan). Bold line indicates the mean across all trailer endings. (*c*) Using a window of 10 seconds on either side of all movie trailer endings, we compared the number of events within this window to a null model where this window is circularly shifted 100 times.

**Scan**	**Title**	**Genre**	**Runtime**
1	Man Up and Go	documentary/emotional	4m20s
1	The First 70	documentary	3m
1	Fixation	documentary/adventure	1m42s
1	The Living	drama	2m
1	SAMSARA	documentary/“unparalleled sensory experience”	1m35s
1	Blood Brother	documentary	2m20s
2	Birdmen	documentary/adventure	3m59s
2	Groomed	drama	1m30s
2	Cold	outdoor/sports	2m
2	Sleepwalkers	drama	2m
2	A Kind of Show	comedy	1m
3	Geofish	documentary/adventure	4m40s
3	The Debut	outdoor/sports	3m23s
3	Dreams of a Life	documentary/mystery	2m10s
3	The Front Man	documentary	2m30s
3	This Is Vanity	drama	1m
4	Planetary	documentary	4m30s
4	Sign Painters	documentary	2m50s
4	Florida Man	documentary/drama	2m
4	The Sleeping Bear	drama	3m40

TABLE S1. **Movies included in each movie scan for the Indiana University dataset.**

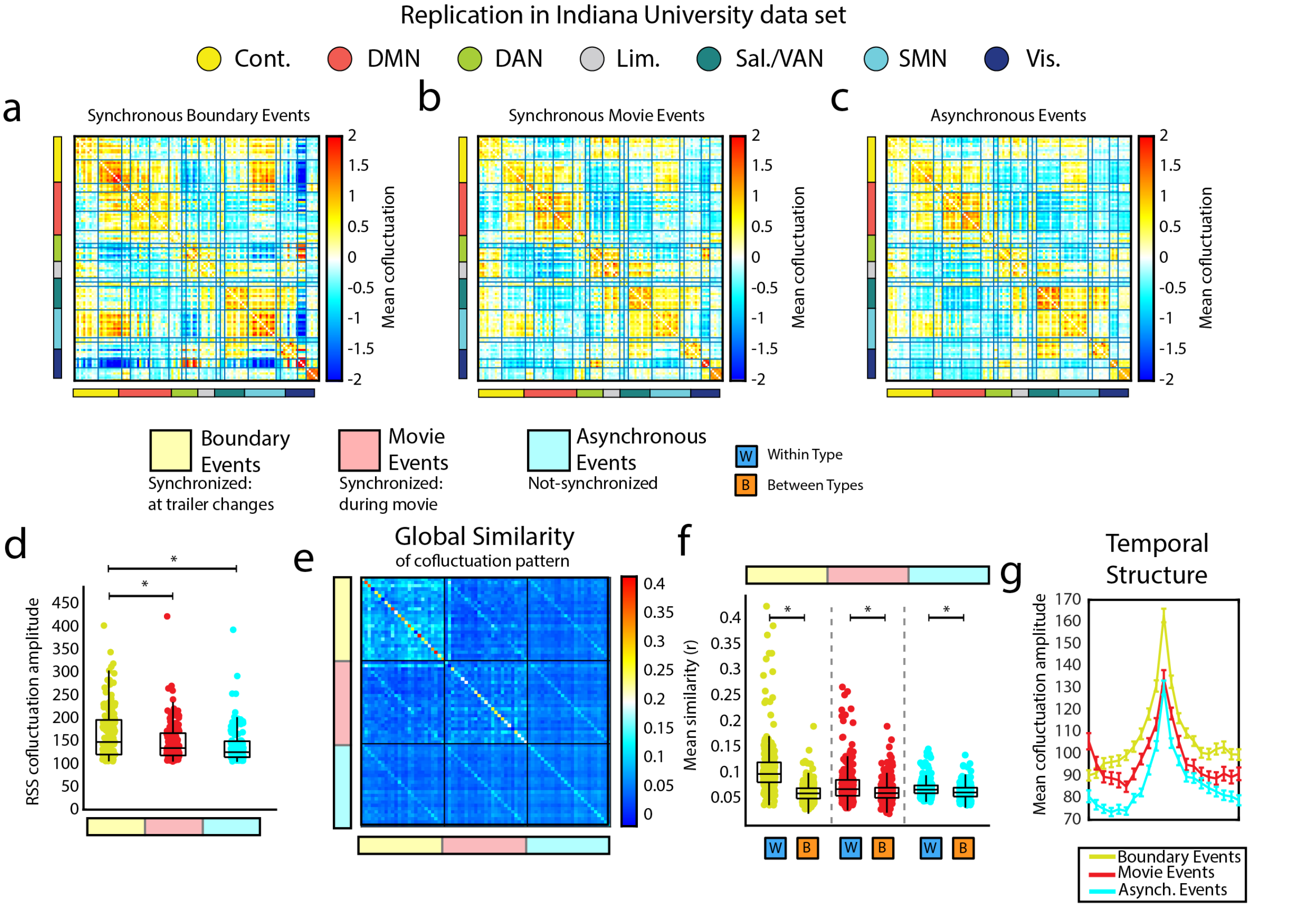

FIG. S2. **Three types of events: replication in Indiana University data set.** (*a-c*) Mean co-fluctuation matrices representing the mean of all events for a given event type. (*d*) Boxplots of the distribution of co-fluctuation amplitudes per event type. (*e*) Global similarity within and between different event types (global similarity is defined in the methods section). (*f*) Boxplots showing the data from the previous figure divided by similarity values within the same type and similarity values between different types. (*g*) Plot showing the mean temporal trajectory of each event type.

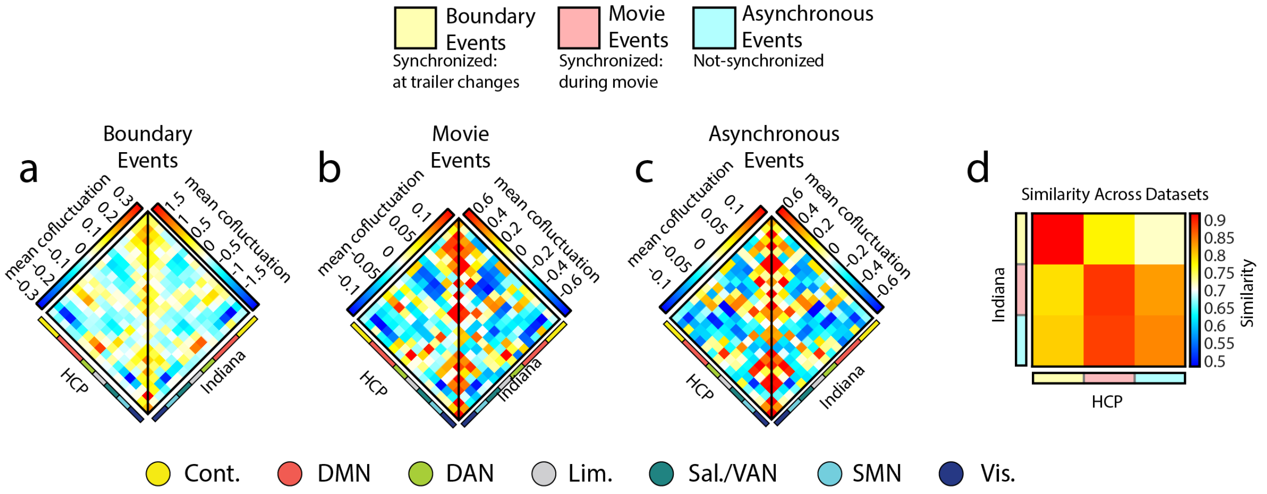

FIG. S3. **Main dataset co-fluctuation patterns for event types are related to the co-fluctuation patterns found in the replication dataset.** (*a*-*c*) System by system mean co-fluctuation patterns across boundary events, movie events, and asynchronous events$_3$ respectively for both data sets (HCP: left; Indiana: Right). (*d*) Across data set similarity of system by system mean cofluctuation matrices for each event type.

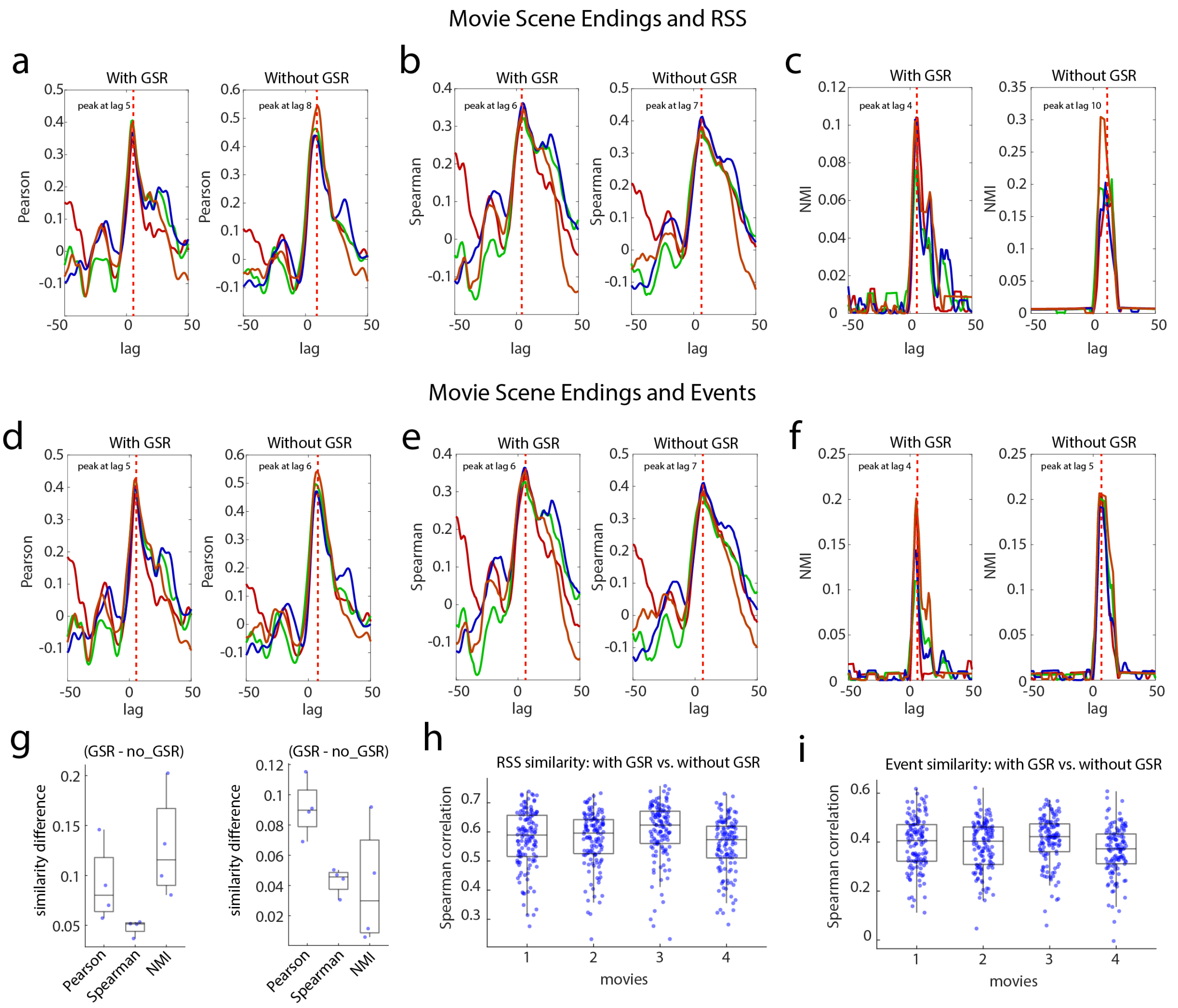

FIG. S4. **Main result with different similarity metrics and with/without global signal regression.** (*a-c*) Time-lagged correlation between HRF convolved movie scene indices and mean RSS (across subjects) using three different similarity metrics (Pearson, Spearman, and normalized mutual information [NMI]). We calculated these time-lagged correlations with and without global signal regression (GSR). All peak correlations are significant ($p < 10^{-15}$). The peak correlation values tend to occur later without global signal regression. (*d-f*) Time-lagged correlation between HRF convolved movie scene indices and mean event time series (across subjects) using three different similarity metrics (Pearson, Spearman, and normalized mutual information [NMI]). We calculated these time-lagged correlations with and without GSR. All peak correlations are significant ($p < 10^{-15}$). The peak correlation values tend to occur later without global signal regression. Importantly, negative values in the movie scene ending indices (induced by convolution with the HRF) were removed for the Spearman correlation analysis as these negative values introduced artifacts in the rank calculation. (*g*) Difference between the peak similarity values of data with GSR and data with no GSR (for all three similarity metrics). Similarity values were always greater for the data with no GSR. (*h*) Boxplots showing the Spearman correlation between RSS time series with GSR and without GSR. (*i*) Boxplots showing the Spearman correlation between event time series with GSR and without GSR.

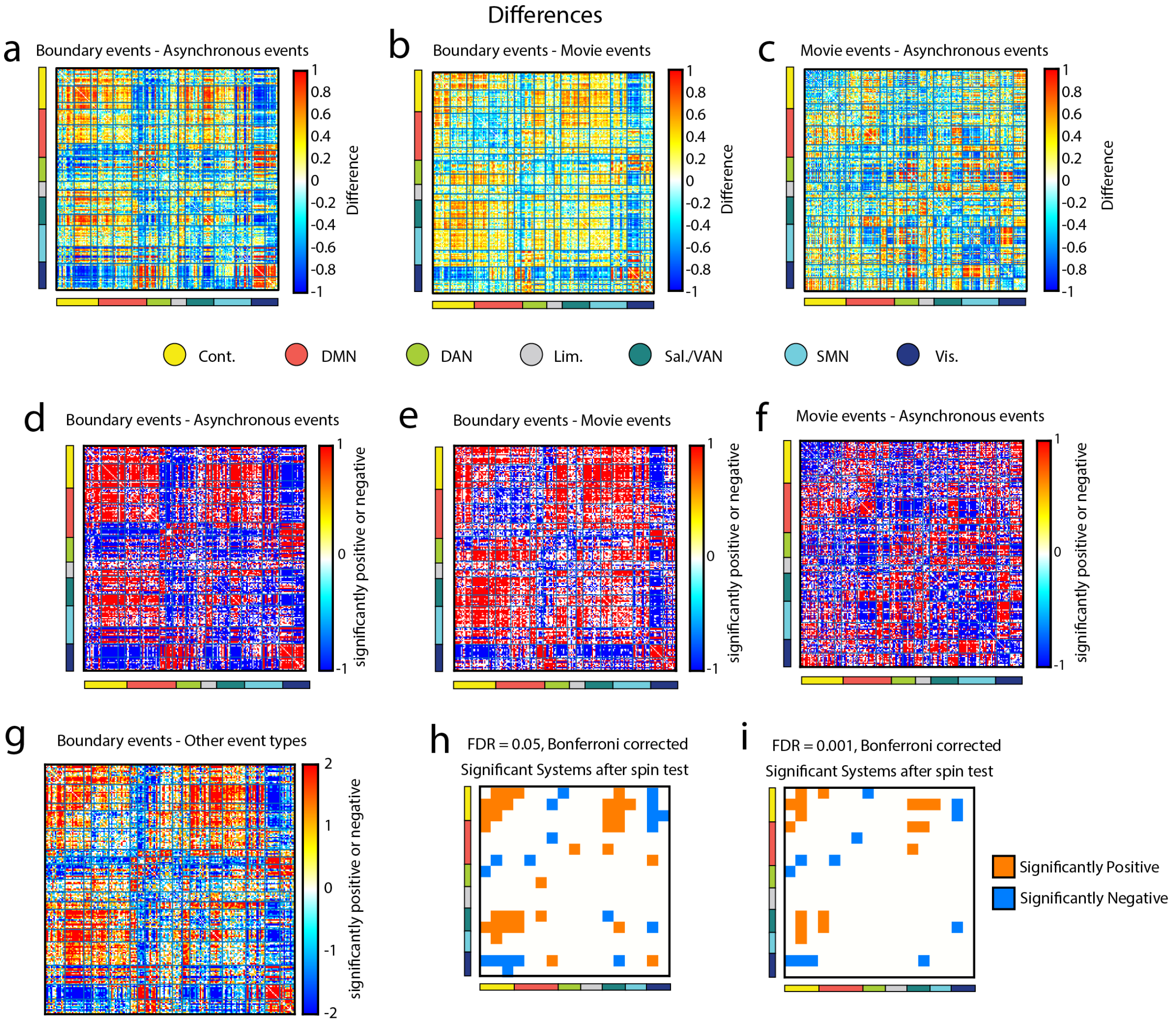

FIG. S5. **Differences between the Event Types** All differences described here are computed using the method detailed in the methods section. (*a*) Difference between boundary events_1 and asynchronous events. (*b*) Difference between boundary events_1 and movie events. (*c*) Difference between movie events_2 and asynchronous events. (*d*) Significantly different edges between boundary events_1 and asynchronous events_3 each colored according to whether the difference was significantly positive or significantly negative. (*e*) Significantly different edges between boundary events_1 and movie events_2 each colored according to whether the difference was significantly positive or significantly negative. (*f*) Significantly different edges between movie events_2 and asynchronous events_3 each colored according to whether the difference was significantly positive or significantly negative. (*g*) Significantly different edges between boundary events_1 and both asynchronous events_3 as well as movie events_2 each colored according to whether the difference was significantly positive or significantly negative. (*h*) Significant system by system edges after running a space-preserving null model (spin test) to test if the significant edges in the previous figure are more concentrated in system by system blocks than we should expect by chance. Here we controlled for multiple comparisons by fixing the false discovery rate to $q = 0.05$. (*i*) Same test as previous figure with a false discovery rate set to $q = 0.001$.

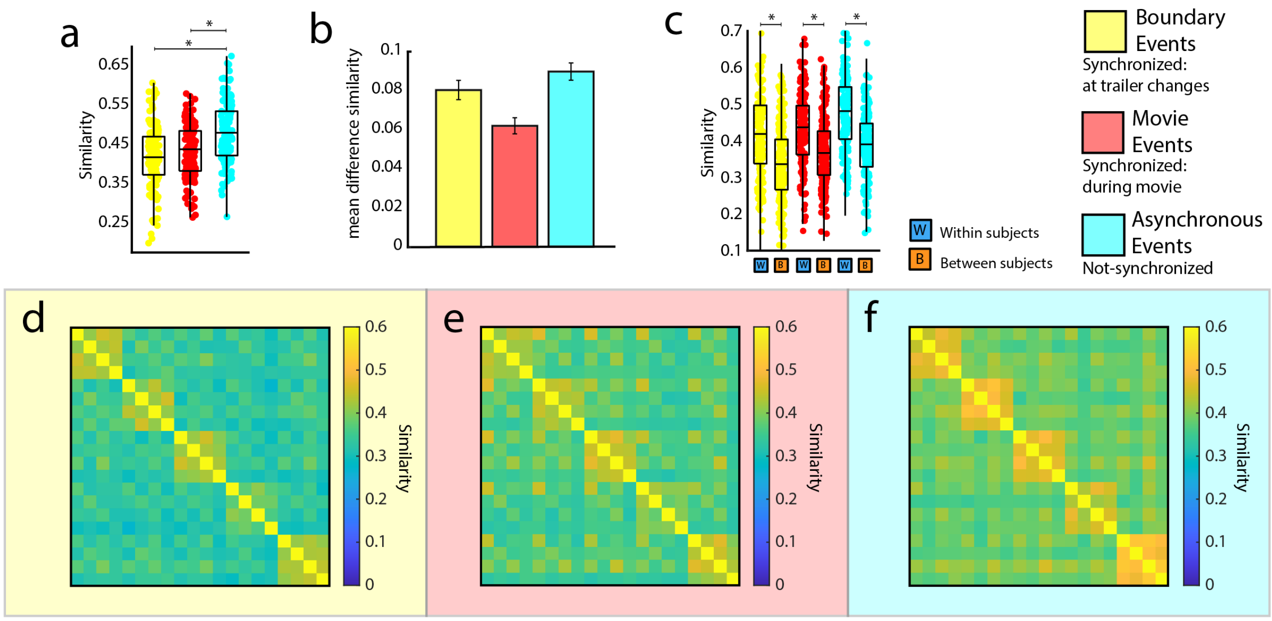

FIG. S6. **Mean co-fluctuation patterns of asynchronous events$_3$ carry more subject-specific information than other event types.** Following research that suggests that events offer increased identifiability in human brain networks [**?**], we explored the identifiability of each event type. In these analyses, we derived mean co-fluctuation patterns for each type of event and for each scan. The different event types occurred at different rates. To ensure that mean co-fluctuation patterns were estimated using similar number of samples, we sub-sampled from the total number of frames assigned to each event type and calculated the mean co-fluctuation pattern using only those frames. (*a*) We find that asynchronous events$_3$ were more similar within subjects compared to boundary or movie events$_2$ (paired-sample t-test $p < 3.37 \times 10^{-13}$). While all event types maintain significant individualized features such that within subject similarity is higher than between subject similarity (*c*; two-sample t-test $p < 10^{-15}$), we find that asynchronous events$_3$ maintain the greatest difference in within versus between-subject similarity (*b*) [Barplots showing the difference between the means of the within-subject similarity distribution and the mean of the between-subject similarity distribution for each event type. Error bars represent the standard error of the difference between the two means.] Panels (*d*-*f*) show similarity matrices representing a set of 5 subjects for each event type. Blocks along the diagonal indicate within-subject similarity across 4 scans. Off-diagonal elements indicate between-subject similarity. In order to display most of the subjects while still maintaining the visual intuition that these plots provide with 5 subjects, we took the mean of these matrices for 25 non-overlapping windows into the full similarity matrix (representing a total of 125 out of the 129 subjects). Notice how the within-subject blocks are strongest in asynchronous events. We also found strong subject-level effects across event types when events are considered individually instead of taking the mean (Fig. S7; synchronous movie events$_2$ did not pass identifiability tests for two scans). To confirm this, we directly compared the within-subject similarity to between-subject similarity for every frame of every event type separately (two-sample t-test $p < 8.66 \times 10^{-4}$). Interestingly, we found that – when considered individually – identifiability was actually greater in boundary events$_1$ (Fig. S7; two-sample t-test $p < 10^{-15}$). This difference in the relative identifiability of event types is likely due to the overall similarity of boundary events$_1$ to one another (recall Fig. **??**d,e). That is, the pattern of co-fluctuation found in asynchronous events$_3$ is more diverse, but in aggregate asynchronous events$_3$ pull together more identifiable information, while on an individual basis more identifiable information can be found in boundary events.

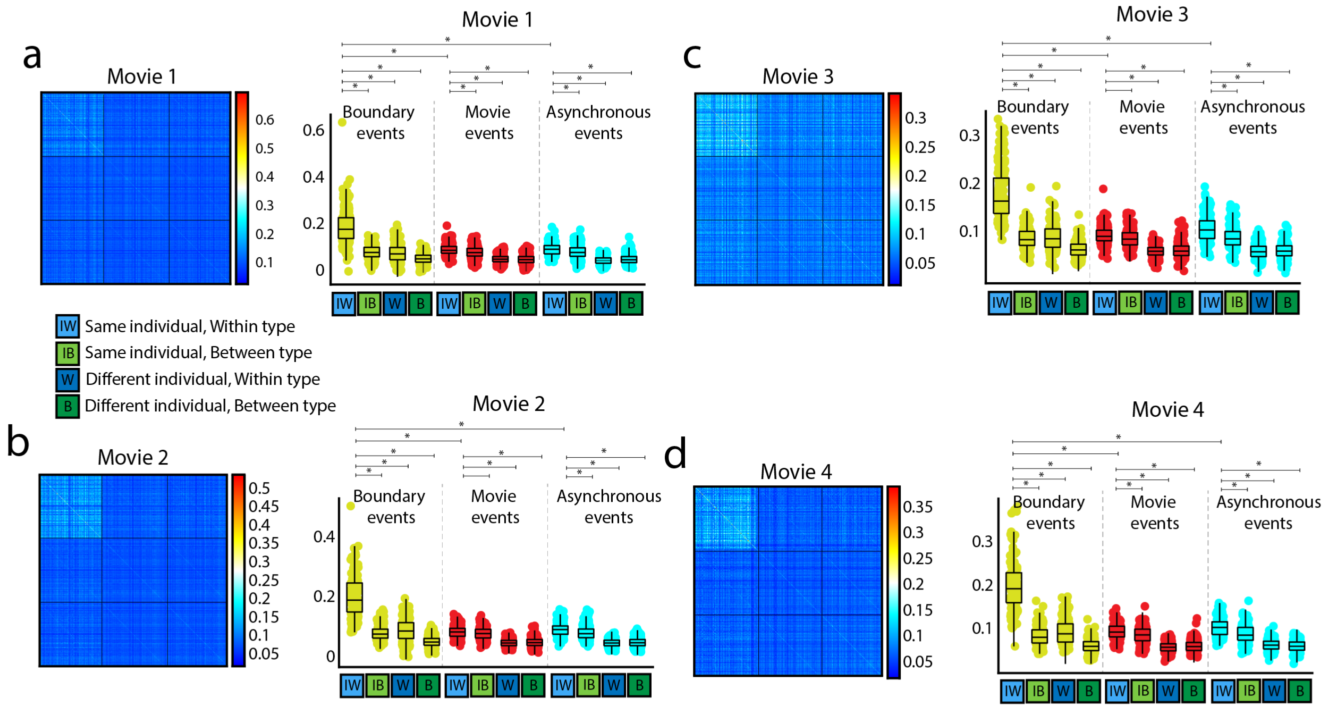

FIG. S7. **Identifiability of the event types when considered frame by frame.** (*a-d*) Each of the following figures show two plots: a similarity matrix and boxplots of these values divided into four categories per event type (same individual-within type, same individual-between type, different individual-within type, and different individual-between type). All p-values below $p < 8.66 \times 10^{-4}$, p-values for boundary events$_1$ $p < 10^{-15}$.

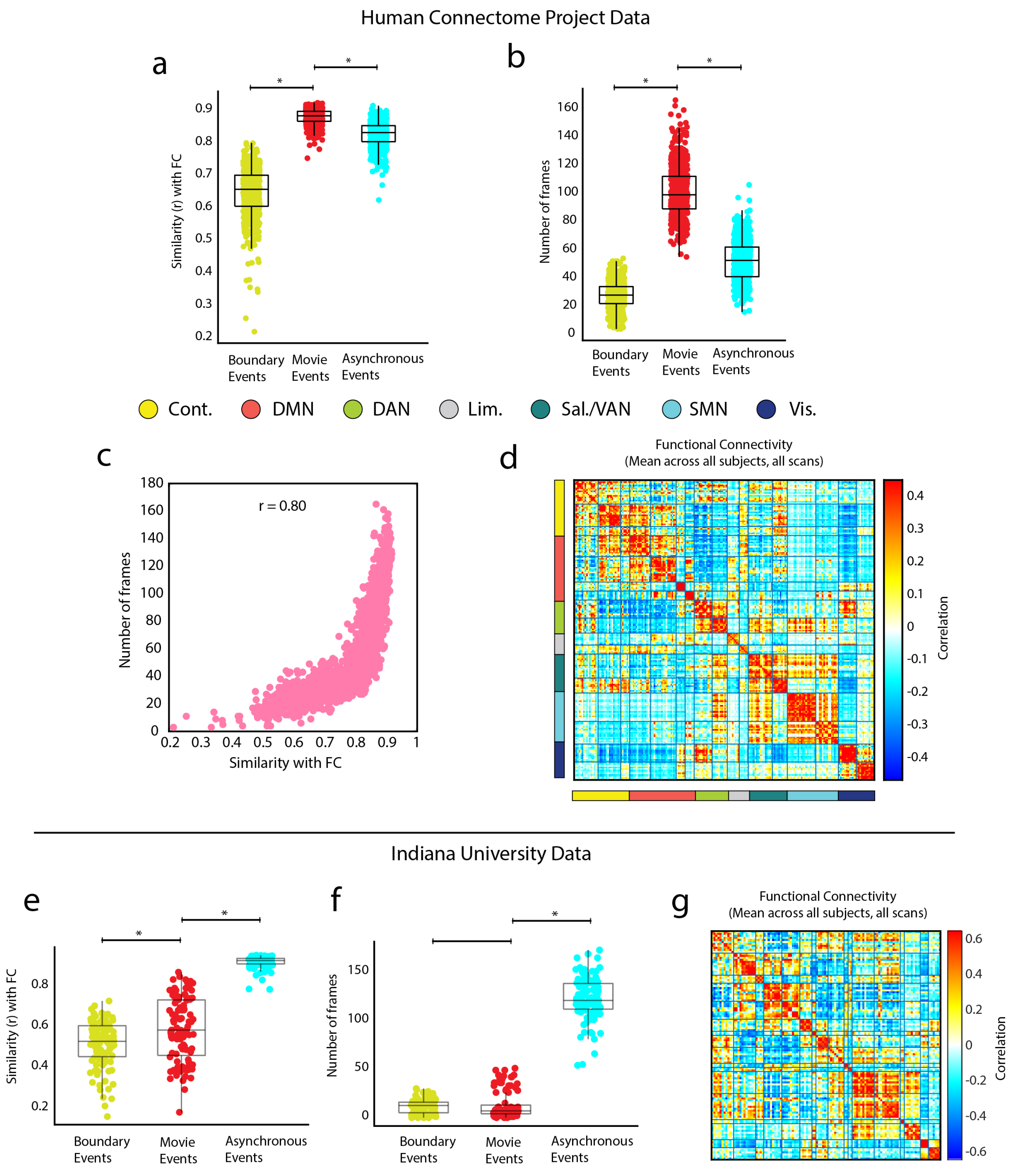

FIG. S8. **movie events$_2$ are the most similar to functional connectivity in HCP data.** (a) Boxplots showing the distribution of correlations between an event type (mean across the participants scan) and FC for the same scan. movie events$_2$ are the most similar to FC (all p-values below $p < 10^{-15}$). (b) Boxplots showing the number of frames assigned to each event type per scan/participant (all p-values below $p < 10^{-15}$). (c) Scatter plot showing the relationship between similarity with FC and number of frames. Similarity with FC appears to be at least partially driven by the number of frames used to compute the mean cofluctuation pattern for an event type. (d) Mean functional connectivity across all scans/participants. (e) Boxplots showing the distribution of correlations between an event type (mean across the participants scan) and FC for the same scan. (f) Boxplots showing the number of frames assigned to each event type per scan/participant. (g) Mean functional connectivity across all scans/participants.

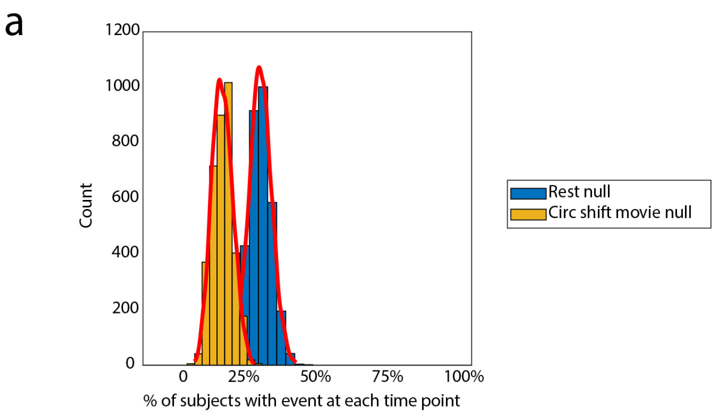

FIG. S9. **Rest-based null model is more conservative than circularly shifted movie event time series.** (*a*) Plot of two null distributions. The rest null was computed as the percentage of subjects with an event at each time point while subjects were at rest. The circularly shifted movie null was computed as the percentage of subjects with an event at each time point. Each participants event time series was circularly shifted to maintain the number and relative timing of events in the actual data, but destroy temporal alignment across subjects. Note that the peak of the rest null distribution estimates that a greater percent of subjects might temporally align events by chance than the circular shift movie null.

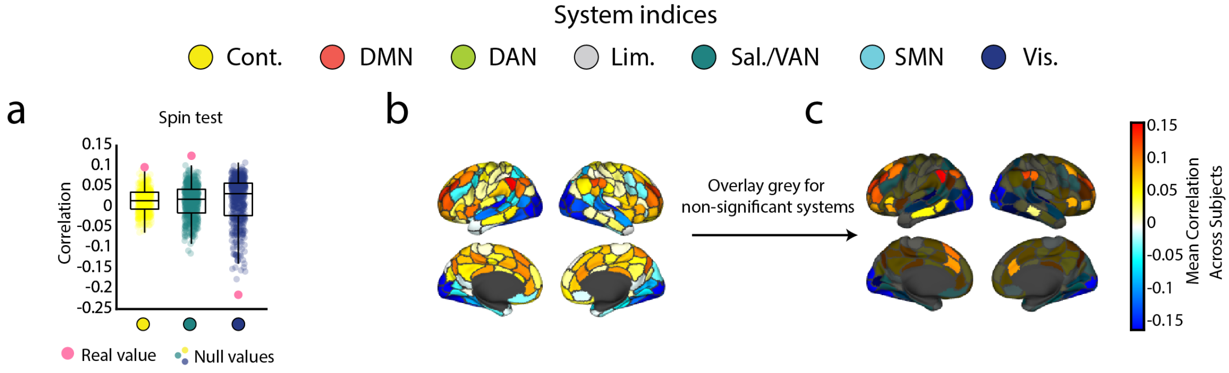

FIG. S10. **Spin test shows that correlations are significantly concentrated in control b, salience b, and central visual systems.** (*a*) We found that in three systems (control b, salience b, and central visual) the mean correlation value between nodal activations in that system and boundary events$_1$ was significantly higher than a null distribution created using a space-preserving permutations test (spin test). (*b*) All correlations plotted to the brain surface. (*c*) Systems that were not significant were overlaid with grey.

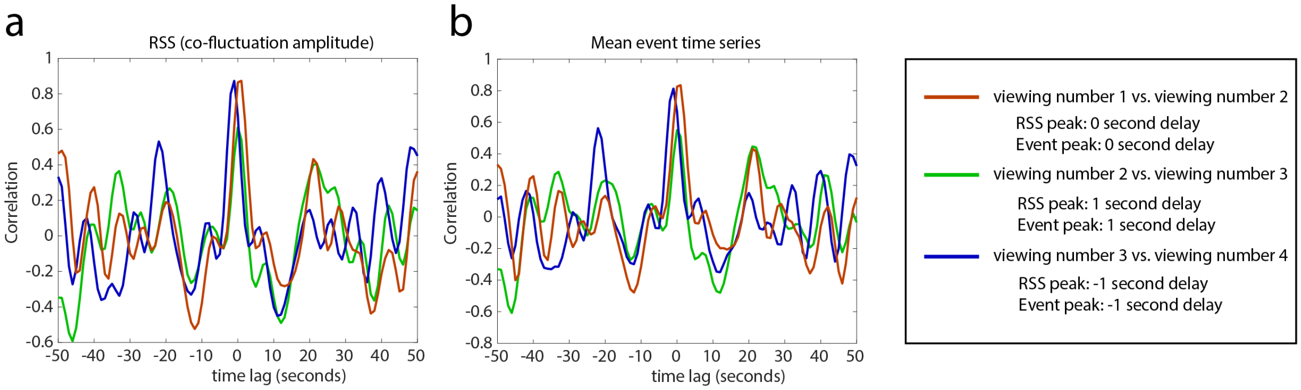

FIG. S11. **RSS and event time series do not show consistent temporal shifts on multiple viewings.** (a) Time-lagged correlations between the mean RSS time series (across subjects) and different viewings of the same movie scene (in reference to the main results shown in Fig. **??**)(b) Time-lagged correlations between the mean event time series (across subjects) and different viewings of the same movie scene. Panel shows correspondence between colored lines and the correlation of different viewings.

.

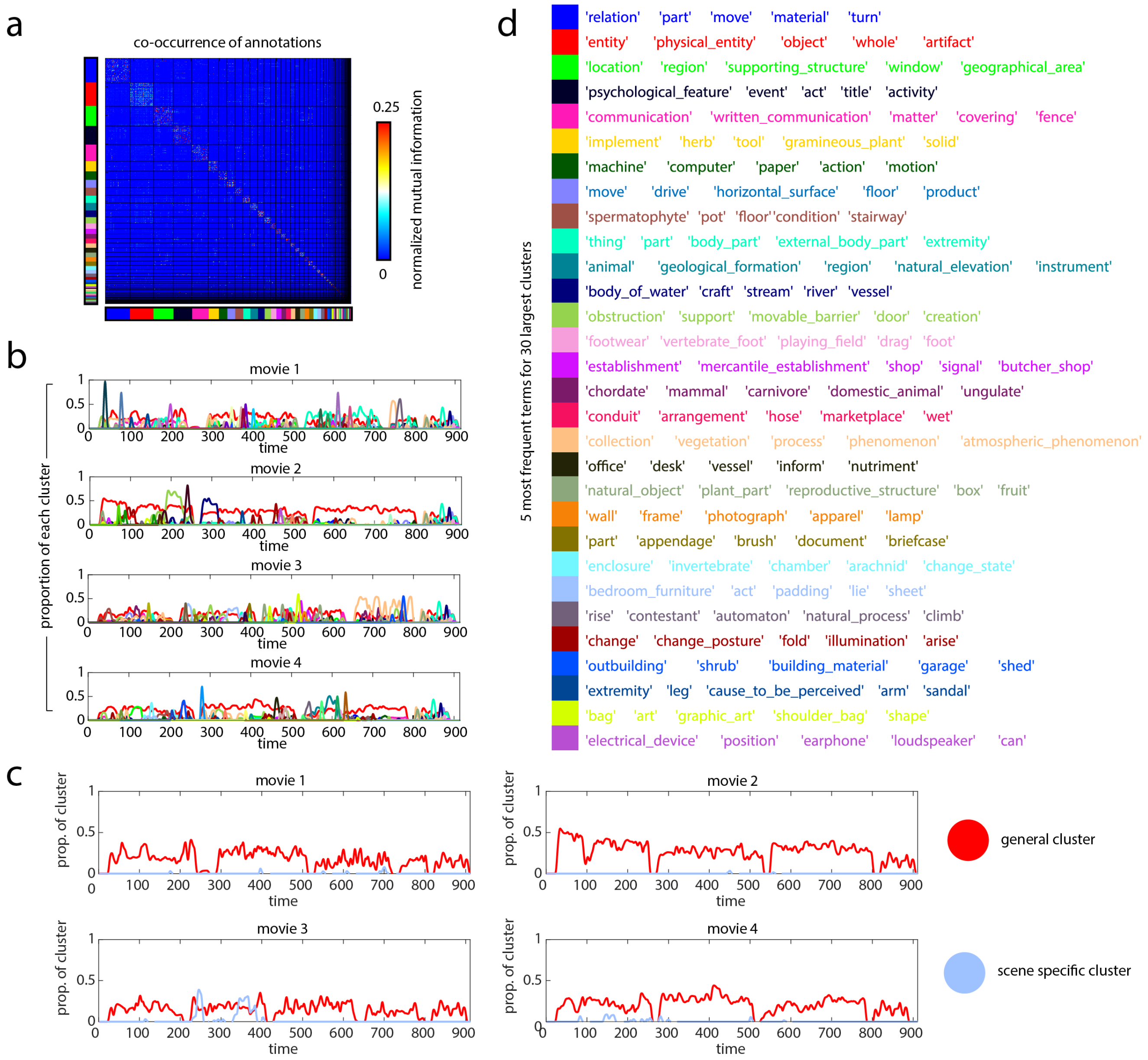

FIG. S12. **Co-occurrence of annotations for all movie scenes in the Human Connectome Project dataset.** (*a*) Co-occurrence matrix where each i by j edge represents the normalized mutual information for all i by j time series of terms. We used consensus clustering with modularity maximization to arrive at a total of 58 clusters (44 non-singleton clusters). Clusters are organized about the diagonal by the number of nodes in each cluster. (*b*) Time series representing the proportion of terms present in each cluster. Time series are color coded to correspond to clusters. (*c*) Here we plot two representative clusters: one that appears to be general to many scenes, and one that appears to be scene specific (mainly occurring during a scene in movie 3). (*d*) A color coded list of the top five most frequent term for the top 30 clusters.
